# The Evolution of Extreme Polyandry in Social Insects: Insights from Army Ants

**DOI:** 10.1371/journal.pone.0105621

**Published:** 2014-08-21

**Authors:** Matthias Benjamin Barth, Robin Frederik Alexander Moritz, Frank Bernhard Kraus

**Affiliations:** 1 Institute of Biology, Department of Zoology, Martin-Luther-University Halle-Wittenberg, Halle (Saale), Germany; 2 DNA-Laboratory, Museum of Zoology, Senckenberg Natural History Collections Dresden, Dresden, Germany; 3 Department of Zoology and Entomology, University of Pretoria, Pretoria, South Africa; 4 Department of Laboratory Medicine, University Hospital Halle, Halle (Saale), Germany; Universidade de São Paulo, Faculdade de Filosofia Ciências e Letras de Ribeirão Preto, Brazil

## Abstract

The unique nomadic life-history pattern of army ants (army ant adaptive syndrome), including obligate colony fission and strongly male-biased sex-ratios, makes army ants prone to heavily reduced effective population sizes (*N*_e_). Excessive multiple mating by queens (polyandry) has been suggested to compensate these negative effects by increasing genetic variance in colonies and populations. However, the combined effects and evolutionary consequences of polyandry and army ant life history on genetic colony and population structure have only been studied in a few selected species. Here we provide new genetic data on paternity frequencies, colony structure and paternity skew for the five Neotropical army ants *Eciton mexicanum*, *E. vagans*, *Labidus coecus*, *L. praedator* and *Nomamyrmex esenbeckii*; and compare those data among a total of nine army ant species (including literature data). The number of effective matings per queen ranged from about 6 up to 25 in our tested species, and we show that such extreme polyandry is in two ways highly adaptive. First, given the detected low intracolonial relatedness and population differentiation extreme polyandry may counteract inbreeding and low *N*_e_. Second, as indicated by a negative correlation of paternity frequency and paternity skew, queens maximize intracolonial genotypic variance by increasingly equalizing paternity shares with higher numbers of sires. Thus, extreme polyandry is not only an integral part of the army ant syndrome, but generally adaptive in social insects by improving genetic variance, even at the high end spectrum of mating frequencies.

## Introduction

Army ants are major arthropod predators and important keystone organisms in tropical and subtropical ecosystems, with a strong impact on the population dynamics of their prey and species community structures [1]–[5]. This is facilitated by a highly specialized combination of traits, the so called army ant adaptive syndrome, which includes group raiding and nomadism [6]–[8]. Several species can establish huge colonies with over a million workers sweeping the forest floor in fan-shaped swarm raids, thereby exhibiting impressive organizational complexity and efficient division of labor [1], [5], [6], [9], [10].

Nonetheless, as typical for the eusocial Hymenoptera (ants, social bees and social wasps), army ants may suffer from constrained effective population sizes (*N*_e_) due to the limited number of reproductive queens and the haploidy of males [11]–[13]. This effect is enhanced by the army ant syndrome, because queens are wingless and can only disperse via colony fission, leading to strongly male-biased sex-ratios [4], [8], [14], which further reduces *N*_e_ and makes army ants susceptible to random drift and deleterious effects of inbreeding [15]. In the vast majority of army ant species, the colonies are headed by a single queen, which is highly polyandrous, mating with about 20 males [16]. Several hypotheses have been put forward to explain the adaptive value of such high degrees of polyandry in social insects, mainly invoking the enhancement of genotypic variance within colonies, but also the potential to increase overall *N_e_* and thus counteract the risk of inbreeding.

The gain in intracolonial genetic variance (“genetic variance” hypothesis) is one of the most prominent hypotheses for the evolution of polyandry in social insects. Genetic variance may support overall colony fitness in various aspects like increasing colony productivity, tolerance to variable environments and pathogen resistance [17]–[22]. Moreover, the presence of many patrilines within a colony may improve worker caste specialization and thus division of labor [23]–[26]. A potential problem for the genetic variance hypothesis at first glance is to explain the evolution of extreme polyandry. This is due to the fact that the genotypic diversity gained with each additional mating drops sharply beyond six to ten effective matings as the average intracolonial relatedness approaches the 0.25 asymptote [27]–[29]. However, at colony level, the synergistic fitness gain of mutualistic interactions between genotypically diverse individuals, known as social heterosis [30], may well increase beyond this threshold and also explain extreme polyandry in concordance with the genetic variance hypothesis. Other hypotheses emphasize the importance of polyandry to overcome limited sperm supply (“sperm limitation” hypothesis) to facilitate huge colony sizes [31]–[34]. Finally, mating with males from many different colonies may increase *N*_e_ and counteract population fragmentation and inbreeding [35]–[37].

However, data on genetic colony structure and paternity frequencies in army ants are so far almost only available for the few intensively studied and easily accessible swarm-raiders of the genera *Eciton* and *Dorylus* (e.g., [16], [23], [35], [36], [38]). The study of Kronauer et al. [16] is so far the only one to compare four distantly related species, concluding that extreme polyandry is ancestral for army ants. Only a single reversion to near monandry (one to two matings) has recently been described for *Neivamyrmex carolinensis*, where colonies are polygynous instead [39], [40]. However, army ant communities in the tropics may comprise more than 20 sympatric species [41], all profoundly differing in their niches and life history [2]–[6], [10]. The majority of these species and their genetic colony structure are still unexplored and may offer important insights into the adaptive value of the army ant mating system.

Here we add paternity data on five Neotropical army ant species, namely *Eciton mexicanum*, *E. vagans*, *Labidus coecus*, *L. praedator* and *Nomamyrmex esenbeckii* (Ecitoninae), and compare them with respect to paternity frequency and genotypic colony composition. The two genera *Labidus* and *Nomamyrmex* were so far neglected in genetic army ant studies, despite their ubiquity and ecological significance [2], [3], [5], [42], [43]. *E. mexicanum* and *E. vagans* resemble their well-studied sister species, the swarm-raider *E. burchellii*, but have considerably smaller and more cryptic colonies [4]–[6], [9], [10]. By deducing queen and male genotypes from worker samples in classical microsatellite DNA analyses, we determine paternity to estimate queen mating frequencies, intracolonial relatedness and paternity skew (distribution of proportional paternity among siring males). Additionally we extract data of four more army ant species from the literature, for comparisons with our own empirical results.

With our data set we test critical predictions made by the various hypotheses for the evolution of extreme polyandry in social insects. First, relatedness analyses within colonies may reveal its potential to counteract inbreeding [36]. Second, by correlating paternity data with colony size we test for the sperm limitation hypothesis. Third, we use paternity skew analyses to test for the genetic variance hypothesis (i.e., for social heterosis). As a high skew can diminish intracolonial genotypic variance gained from polyandry by shifting the average intracolonial relatedness towards that of effective monandry [19], [34], [44], [45], queens are expected to equalize the paternity distribution among sires increasingly with higher paternity frequencies to maximize variance gains. In the absence of such a selection pressure male (sperm) competition should lead to monopolization of paternity [22], [34], [46], [47]. Consequently, Jaffé et al. [47] found a negative association of paternity skew and paternity frequency over a large paternity gradient across polyandrous Hymenoptera. However, they did not focus on extremely polyandrous species, while Kronauer et al. [16] hypothesized selective neutrality for the exact number of mates in this case. The present study is, to our knowledge, the first attempt to test this prediction and to show whether an association of paternity skew and frequency is maintained (i.e., genetic variance remains important) at the high end spectrum of polyandry within the clade of ancestrally extremely polyandrous army ants.

## Materials and Methods

### Sampling and genotyping

Sampling was carried out in Chiapas, Mexico, near the city of Tapachula at three different locations with a maximal distance of 18 km between them (Table 1): Cacahoatán (15°0′19 N, 92°10′17 W), Tapachula (14°55′58 N, 92°17′01 W) and Tuxtla Chico (14°58′25 N, 92° 9′34 W). Sampling permits were authorized by ECOSUR (El Colegio de la Frontera Sur, Unidad Tapachula) and the Agricultural Research Centre Rosario Izapa in Tuxtla Chico. The sampling did not involve endangered or protected species. Workers of the five study species were collected from columns and immediately stored in 95% ethanol and kept at –20°C until further laboratory processing: *E. mexicanum* s. str. Roger (3 colonies, *n* = 400 workers), *E. vagans angustatum* Roger (1 colony, *n* = 94), *L. coecus* Latreille (1 colony, *n* = 94), *L. praedator* s. str. Smith (3 colonies, *n* = 294) and *N. esenbeckii wilsoni* Santschi (2 colonies, *n* = 94). Species were identified using the key of Watkins [43].

**Table 1:** Sampling locations, paternity frequencies, genetic heterozygosities and paternity skew of up to three colonies of the five Neotropical army ants *E. mexicanum* (*Em*), *E. vagans* (*Ev*), *L. coecus* (*Lc*), *L. praedator* (*Lp*) and *N. esenbeckii* (*Ne*).

Colony	Location	*n*	*k*_obs_	*k*_est_	*m*_e_	*H*_O_	*H*_S_	*B*
*Em*1	Cacahoatán	115	18	18.03	15.07	0.68	0.68	0.0106*
*Em*2	Cacahoatán	125	12	12.00	10.32	0.68	0.74	0.0133*
*Em*3	Tuxtla Chico	154	20	20.01	18.02	0.65	0.68	0.0054*
*Em* over all		394	16.67±1.04	16.68±1.04	13.71±1.10	0.67±0.02	0.70±0.03	0.0098*
*Ev*1	Tuxtla Chico	94	22	22.31	19.18	0.90	0.86	0.0064*
*Lc*1	Tapachula	93	11	11.00	8.10	0.70	0.72	0.0319*
*Lp*1	Cacahoatán	104	26	26.48	25.51	0.77	0.77	0.0006
*Lp*2	Tapachula	108	18	18.04	15.58	0.86	0.82	0.0084*
*Lp*3	Tuxtla Chico	79	26	27.25	22.33	0.81	0.81	0.0059
*Lp* over all		291	23.33±1.15	23.92±1.28	20.25±1.27	0.81±0.05	0.80±0.03	0.0050*
*Ne*1^1^	Cacahoatán	8	5	6.01	5.76			
*Ne*2	Tapachula	80	7	7.00	6.01	0.76	0.83	0.0230*

*n*, number of genotyped workers that have been used for paternity deduction, after excluding workers with less than three unambiguously amplified loci (*Em*: 6, *Ev*: 0, *Lc*: 1, *Lp*: 3, *Ne*: 6); *k*_obs_, observed paternity frequency (number of deduced male genotypes which have been used together with the deduced queens for further analyses); *k*_est_, estimated paternity frequency, corrected for sample size; *m*_e_, effective paternity frequency [55]; *H*_O_ and *H*_S_, observed and expected heterozygosity; *B*, *B*-index of paternity skew [62]. Over all indicates sums for *n*, arithmetic means ± SE from jackknifing over colonies for *k*_obs_ and *k*_est_, harmonic means ± SE for *m*_e_, arithmetic means ± SD for *H*_O_ and *H*_S_.

*significant deviation from zero (Bonferroni adjusted 5% level of simulated *p*-values).

1 For colony *Ne*1 no heterozygosity and paternity skew was estimated due to a low sample size and a relatively high non-sampling error of patrilines (∼20%), so that *k*_obs_ and *m*_e_ are potentially underestimated.

We screened two existing primer sets (Eb and Dmo), originally developed for the army ants *E. burchellii* and *Dorylus molestus* [48], [49] for cross-amplification in all our five study species. Since only up to four loci were polymorphic in *Labidus* and *Nomamyrmex*, we developed five additional microsatellite primers (Lp2, Lp4, Lp14a, Lp30 and Lp38; GenBank accession numbers KF969232 to KF969236; Table S1) for *L. praedator*, following the microsatellite isolation protocol of Glenn and Schable [50]. These newly developed primers were also tested for cross-amplification resulting in a total of 14 microsatellite loci (Table S2) that individually all were polymorphic for at least one of the five tested species. Worker DNA was extracted from ant legs, following a 5% Chelex protocol [51] and amplified in standard PCR cocktails at an annealing temperature of *T*_a_ = 54°C (51°C for DmoD). Allele calling and scoring was performed using the Fragment Profiler software (Amersham Biosciences), and double checked by eye. DNA amplification and genotyping was repeated for 10% of the samples confirming the initial allele scoring. See Protocol S1, Tables S1 and S2 for further details of the microsatellite development and cross-species testing.

### Paternity frequency analysis

Queen and male genotypes were deduced from the worker genotypes by Mendelian inference [52]. If a worker shared the same alleles as its heterozygous queen at a given locus, so that two patrilines were possible at that locus, workers were assigned in a way to minimize the number of patrilines (parsimonious). Workers with less than three unambiguously amplified loci were excluded (Tables 1 and S3). Taking advantage of the polyandrous mating system in army ants we used the inferred males and queens as a population sample from which we estimated the population wide allele frequencies with the program FSTAT 2.9.3 [53]. To obtain a diploid input file, males were entered as homozygote diploids and queens were duplicated. These allele frequencies were also used to confirm our manual queen and male deduction in the program MATESOFT version 1.0 [54]. The non-detection error for patrilines, i.e., the probability to obtain two males with the same allele combination at all loci [34], [45] was low for all species (2.2×10^–9^ to 2.3×10^–2^; Table S3). Therefore, the number of deduced patrilines was taken as the observed number of sires a queen mated with, i.e., the observed paternity frequency (*k*_obs_). Correcting for the sample size dependent number of potentially unsampled patrilines (non-sampling error, [45]) we added the expected zero frequencies from fitted Poisson distributions to *k*_obs_ to achieve an estimate of the actual paternity frequencies of the queens (*k*_est_). Additionally, we estimated the sample size corrected genetically effective paternity frequency (*m*_e_), which accounts for potential paternity skew [55].

### Population structure and relatedness

Nei’s observed and expected heterozygosities (*H*_O_ and *H*_S_ [56]) were calculated in FSTAT for all species. For *H*_O_ we used the worker genotypes of one randomly chosen worker per patriline to reduce an allele frequency bias from related workers (though some bias is still expected from the shared queen alleles of workers of the same colony). The unbiased deduced queen and male genotypes were used for *H*_S_. Likewise, we used the worker genotypes to test for Hardy-Weinberg-Equilibrium, and the queen and male genotypes to test for linkage disequilibrium of loci in GENEPOP on the Web, using standard settings [57]. For the two species for which more than two colonies were available (*E. mexicanum* and *L. praedator*), queen and male genotypes were also used in FSTAT to estimate *F*_ST_ values for population sub-structuring [58], treating colonies as sub-populations. Moreover, the inbreeding coefficient was estimated from Nei’s heterozygosities: *F*_IS_ = 1– *H*_O_/*H*_S_ [56].

To assess the significance of inbreeding from the within colony relatedness, *E. mexicanum* and *L. praedator* were also analyzed with the program RELATEDNESS 5.0.8 [59] in a similar way as described for other army ants [16], [35], [36]. The average pairwise regression genetic relatedness was estimated between queens and their mates (*r*_qm_), among the males of each queen (*r*_mm_) and among the workers (*r*_ww_). In the absence of inbreeding the latter should not differ significantly from the expected pedigree relatedness, *g*_ww_ = 0.25+0.5/*m*_e_ [60], where *m*_e_ is the uncorrected effective paternity frequency from Starr [61]. Single-sample *t*-tests were used to test this relationship per colony (Bonferroni adjusted for multiple comparisons) and over all colonies. Likewise, *r*_qm_ and *r*_mm_ were tested against zero, to check whether queens mated with unrelated males and males were unrelated among themselves, respectively.

### Paternity skew analyses within species

Two different indices were used with two different data sets to estimate intracolonial paternity skew. The first data set consisted of our five study species plus data of six *E. burchellii* colonies from Jaffé et al. [23]. For these six species raw data on per-queen paternity distribution among patrilines were available and were used to quantify and test paternity skew with the binomial *B*-index. This is considered the most robust paternity skew index [62], [63] and was calculated using the program SKEW CALCULATOR 2003 (https://www.eeb.ucla.edu/Faculty/Nonacs/PI.html, [63]). The *B*-index ranges from –1 (even paternity among all males) to 1 (monopoly by one male), with zero indicating a random paternity skew. SKEW CALCULATOR tests for deviation from random skew per species and colony based on one-tailed *p*-values from 1,000 simulations.

**Figure 1:**
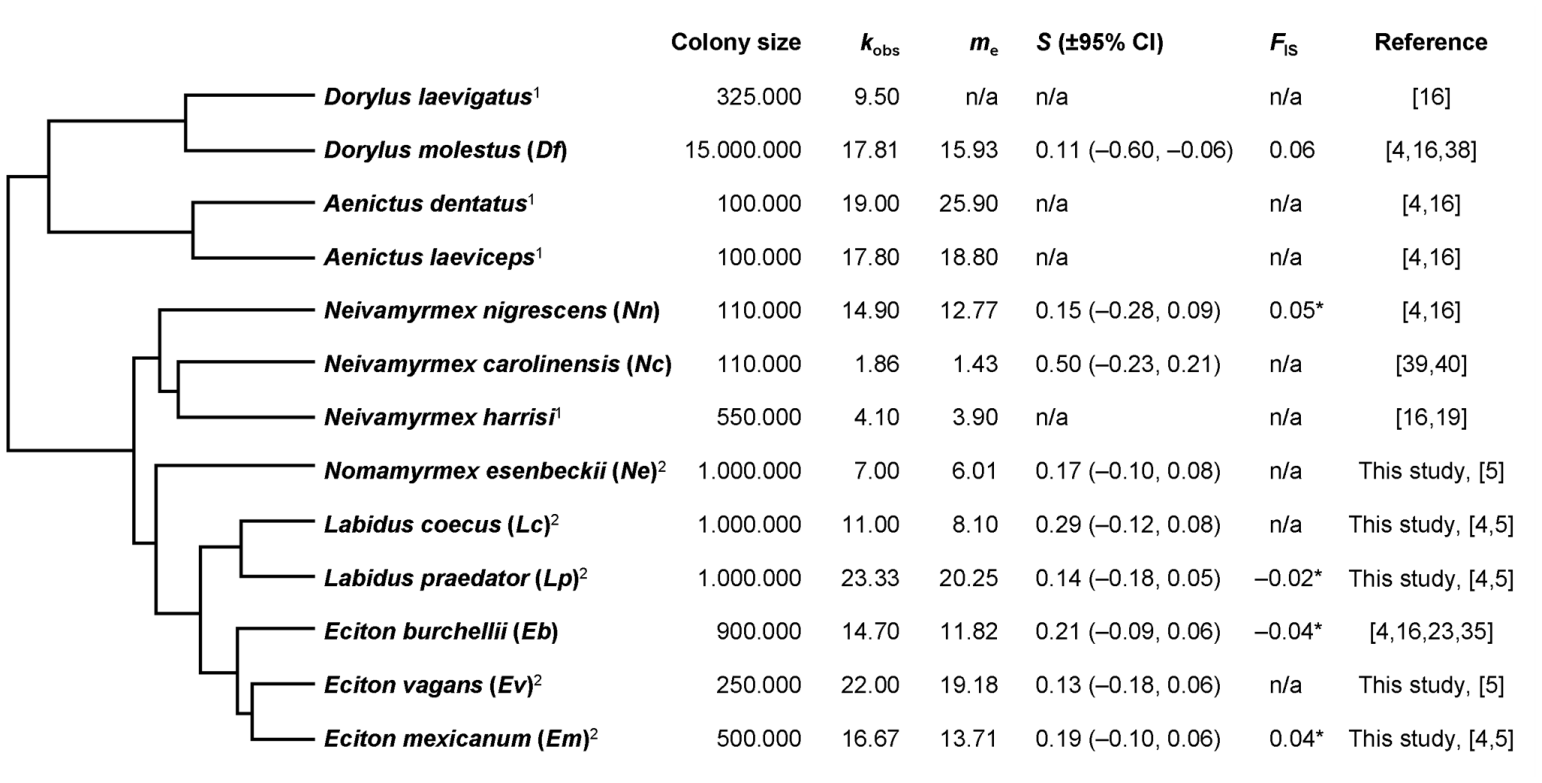
Phylogenetic tree (following Brady [7]) of all 13 army ant species for which paternity data is available so far. Data on colony size, observed and effective paternity frequency (*k*_obs_ and *m*_e_), effective-number-index of paternity skew (*S*) and inbreeding coefficient (*F*_IS_) are given next to the tree. Colony size is represented by the average approximation of the number of workers in normal mature colonies as given in the literature. *k*_obs_ and *m*_e_ are the means given in this study or in the literature, from which *S* was calculated [47], [64]. Mean *F*_IS_ is also taken from this study or the literature (asterisks mark significance). Species abbreviations, as used in the following figure legends, are given in brackets behind species names. n/a, data not available.^ 1^ Data on these species are listed for the sake of completeness, but have not been used in this study because *m*_e_ estimates were either not published, higher than the respective *k*_obs_, or assumed to be underestimated [16], so that in these cases the *S*-index could not be calculated or would have been biased. ^2^ Paternity and inbreeding data on these species are the result of this study.

For the second data set we added paternity data of three more army ant species from the literature (*D. molestus*, *Neivamyrmex nigrescens* and *N. carolinensis*) to our first data set (Figure 1). As for the latter three species no raw paternity data was available to determine the *B*-index, we instead used the published *k*_obs_ and *m*_e_ values to calculate the effective-number-index of paternity skew (*S*-index), with *S* = (*k*_obs_–*m*_e_)/(*k*_obs_–1) [64], for all nine species in the second data set, similar to Jaffé et al. [47]. The *S*-index ranges from 0 to 1, representing even or monopolized paternity, respectively. This index, however, does not account for random paternity skew which is always expected from stochastic effects, such as sampling biases. Therefore, we determined case-specific confidence intervals of randomly expected *S*-values for each species in standard spreadsheet based Monte Carlo simulations [63], [64]. To do so, we calculated *S*-values in such a way that each worker of a given colony had the same probability of belonging to any of the respective observed patrilines, and iterated this protocol 3,000 times, as described in detail in Jaffé et al. [47]. Observed *S*-values were considered significant if outside of the randomly expected confidence limits.

### Paternity skew analyses among species

Both data sets were tested for an association of paternity skew and paternity frequency by applying a phylogenetically corrected generalized least-squared (GLS) regression, based on a maximum likelihood approach [65], [66], with *k*_obs_ as predictor of *B* and *S* skew indices over all species of the respective data set. To do so, the phylogenetic information from Brady [7] was used with our data in the program BayesTraits (http://www.evolution.reading.ac.uk/SoftwareMain.html [65]). According to the genetic variance hypothesis, this association should be negative if queens equalize paternity among sires to maximize colony level fitness gained from mating multiply, so that paternity skew decreases with increasing *k*_obs_ [47]. Furthermore, we also used colony size (Figure 1) as predictor of *k*_obs_ and the *S*-index, respectively, in GLS regressions over all nine species to test the sperm limitation hypothesis. Regression slopes were tested for deviation from zero using likelihood ratio tests.

As paternity skew (*S*-index) estimations in the second data set depend on *k*_obs_, an association between these two variables is expected *a priori*. To test for a deviation from this null expectation, we determined case-specific confidence limits of slopes from Monte Carlo simulations by running the GLS model 100 times with 100 different sets of randomly chosen *S*-values from the first simulations of all nine species (see Jaffé et al. [47]). Negative *S*-values, which are not defined but may occur during the simulations if *m*_e_ exceeds *k*_obs_, were conservatively treated as zero skew. Prior to GLS analyses the not normally distributed variables, paternity skew and colony size, were normalized using log (x+1) (accounting for zero skews) and simple log (x) transformations, respectively.

Differences in the degree of paternity skew among species can also be analyzed by testing paternity distributions among patrilines for deviation from homogeneity in contingency tables. Therefore, we used our first data set in a correspondence analysis in STATISTICA 8.0 [67], similar to Schlüns et al. [68]. Absolute patriline frequencies and the species were entered as categorical variables in rows and columns, respectively. The proportional contribution of each species to the deviation from homogeneity of the paternity skew is represented by relative inertia resulting from *χ* ^2^-statistics.

## Results

### Genotypes and paternity frequencies

The primer characteristics of the five new *L. praedator* microsatellites, as well as all inferred queen and male genotypes are given in Tables S1 and S3, respectively. Locus Lp14a revealed homozygote excess at several alleles in *E. mexicanum*, suggesting the occurrence of one or more null alleles, and was therefore excluded from the analyses for this species. In the end, we used ten loci for *E. mexicanum*, eleven for *E. vagans*, three for *L. coecus*, nine for *L. praedator,* and six for *N. esenbeckii* (Table S2).

In all colonies, except for the *E. mexicanum* colony *Em*1, a single queen genotype explaining all worker genotypes could be inferred with 100% support in MATESOFT. For colony *Em*1 all but two workers could be explained by one queen, for which two different genotypes were possible. We thus used the queen genotype with the higher probability in MATESOFT (78%) and a more parsimonious number of patrilines. The remaining two unassigned workers were excluded for having possibly originated from a second queen as it might occur after recent colony fission.

All here analyzed queens were highly polyandrous with observed paternity frequencies (*k*_obs_) ranging from 5 to 26 siring males (Table 1). Sample sizes were sufficient for all colonies since *k*_obs_ did not differ notably from the sample size corrected estimated paternity frequencies (*k*_est_, 6.01 to 27.25), except for *Ne*1, which was excluded from the following paternity skew analyses because of potential underestimation of the paternity frequency (see Table 1). The genetically effective mating frequencies (*m*_e_) ranged from 5.76 to 25.51 per colony and were lower than *k*_est_ throughout, indicating potential paternity skews [34], [55].

### Population structure and relatedness

Observed heterozygosities (*H*_O_) were high for all species (0.67 to 0.90) and within the range of expected heterozygosities (*H*_S_, 0.68 to 0.86) over all loci (Table 1), suggesting no further homozygote excess after removal of null alleles. All species were in Hardy-Weinberg-Equilibrium over all loci and colonies (Fisher’s exact tests; *E. mexicanum*: *χ* ^2^ = 73.57, *df* = 60, *p* = 0.11; *E. vagans*: *χ* ^2^ = 16.32, *df* = 22, *p* = 0.80; *L. coecus*: *χ* ^2^ = 1.61, *df* = 6, *p* = 0.95; *L. praedator*: *χ* ^2^ = 47.04, *df* = 54, *p* = 0.74; *N. esenbeckii*: *χ* ^2^ = 4.65, *df* = 24, *p*>0.99). Linkage was only detected in *L. praedator* between two to four loci pairs per colony (Bonferroni adjusted *p*-values). However, as different loci pairs were linked in different colonies and no linkage was detected in the other species, the apparent linkage may actually reflect some degree of relatedness among queen’s mates in *L. praedator*, and we assume no physical linkage between loci.

For *E. mexicanum* and *L. praedator* genetic population sub-structuring in the sampling area was weak but significantly larger than zero (one-tailed *t*-tests; *E. mexicanum*: *F*_ST_ = 0.019±0.009 SE from jackknifing over loci, *n* = 56 chromosomal sets, *t* = 2.11, *df* = 9, *p* = 0.03; *L. praedator*: *F*_ST_ = 0.069±0.023, *n* = 76, *t* = 2.96, *df* = 8, *p* = 0.01). Likewise, the mean population inbreeding coefficient was also slightly positive for *E. mexicanum* (*F*_IS_ = 0.039±0.006 SE, *t* = 6.59, *df* = 9, *p*<0.001), but slightly negative in *L. praedator* (*F*_IS_ = –0.016±0.003, *t* = 5.71, *df* = 8, *p*<0.001).

In *E. mexicanum* the average regression genetic relatedness among workers (*r*_ww_ = 0.251±0.030 SE from jackknifing over loci) did not significantly differ from the pedigree relatedness (*g*_ww_ = 0.290) over all colonies (*t* = 1.31, *p* = 0.11). The overall average queen to mate relatedness was slightly negative, though not significantly different from zero (*r*_qm_ = –0.059±0.035, *t* = 1.7, *p* = 0.06). Also the relatedness among males did not significantly differ from zero (*r*_mm_ = 0.004±0.012, *t* = 0.37, *p* = 0.36). In colony *Em*2 *r*_ww_ = 0.199±0.034 was significantly lower than *g*_ww_ = 0.302, (*t* = 3.00, *p*<0.01), suggesting outbreeding. Also in *L. praedator r*_ww_ (0.326±0.036) and *g*_ww_ (0.280) were not significantly different (*t* = 1.29, *p* = 0.12) and the queen to mate relatedness did not differ from zero (*r*_qm_ = 0.017±0.026, *t* = 1.7, *p* = 0.27). The overall among male relatedness (*r*_mm_ = 0.041±0.018) was close to zero though significantly positive (*t* = 2.23, *p* = 0.03). See Table 2 for relatedness data.

**Table 2:** Relatedness estimates for three colonies of *E. mexicanum* and *L. praedator* from Chiapas, Mexico.

Colony	*g*_ww_	*r*_ww_	*r*_qm_	*r*_mm_
*Em*1	0.287	0.252±0.048	–0.035±0.077	0.029±0.024
*Em*2	0.302	0.199±0.034*	–0.153±0.116	–0.035±0.018
*Em*3	0.281	0.293±0.050	–0.011±0.055	0.006±0.011
*Em* over all	0.290	0.251±0.030	–0.059±0.035	0.004±0.012
*Lp*1	0.274	0.403±0.079	0.104±0.070	0.051±0.037
*Lp*2	0.286	0.272±0.036	–0.074±0.052	0.035±0.009*
*Lp*3	0.278	0.292±0.041	–0.013±0.045	0.036±0.014*
*Lp* over all	0.280	0.326±0.036	0.017±0.026	0.041±0.018*

*g*_ww_, pedigree relatedness [60], [61]; *r*_ww_, *r*_qm_ and *r*_mm_, average regression genetic relatedness among workers, of queens to their mates and among siring males (± SE from jackknifing over loci).

*significant deviation from zero for *r*_qm_ and *r*_mm_, or significant deviation of *r*_ww_ from *g*_ww_ (Bonferroni adjusted 5% level).

### Paternity skew analysis

Paternity skew was significant in both data sets, with mean *B*-values higher than zero (Table 1) and effective number indices *S* above randomly expected 95% confidence limits (Figure 1) for all tested species. Only for the two *L. praedator* colonies *Lp*1 and *Lp*3 paternity sharing of patrilines did not differ from a random skew (Bonferroni adjusted *p*-values). However, mean *B*-values over all *L. praedator* colonies indicate significant paternity skew, which is also confirmed by the *S*-index.

**Figure 2:**
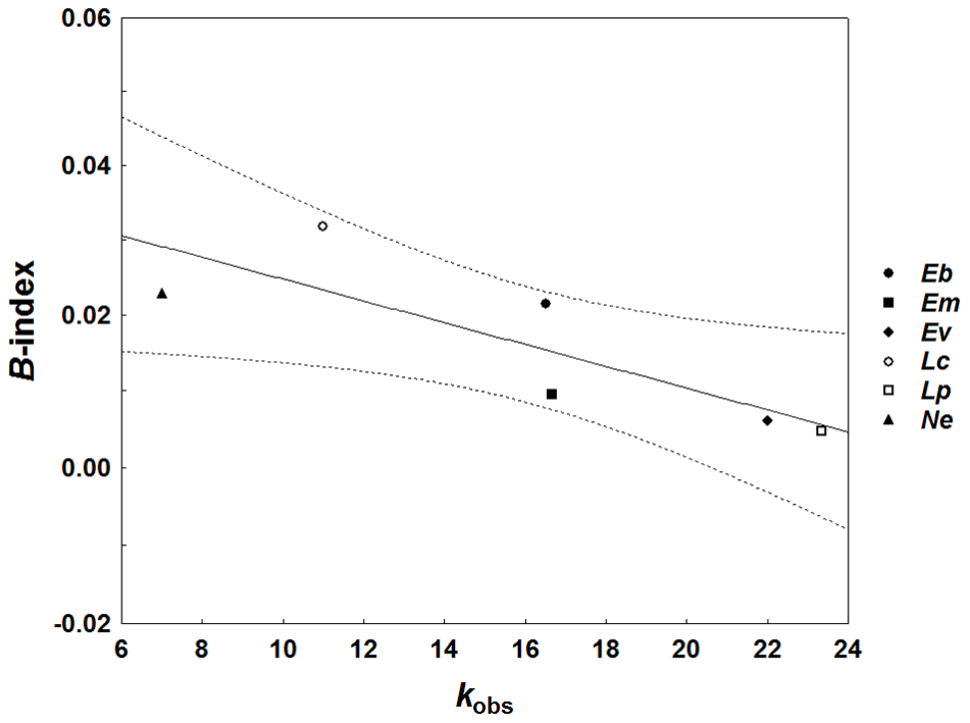
Association of mean paternity skew (*B*-index) and mean observed paternity frequencies (*k*_obs_) across six Neotropical army ant species (represented by different symbols). The solid line indicates the slope of a phylogenetically corrected GLS regression (*b* = –0.001, *R* ^2^ = 0.74, *p* = 0.008) and dashed lines the 95% confidence interval.

**Figure 3:**
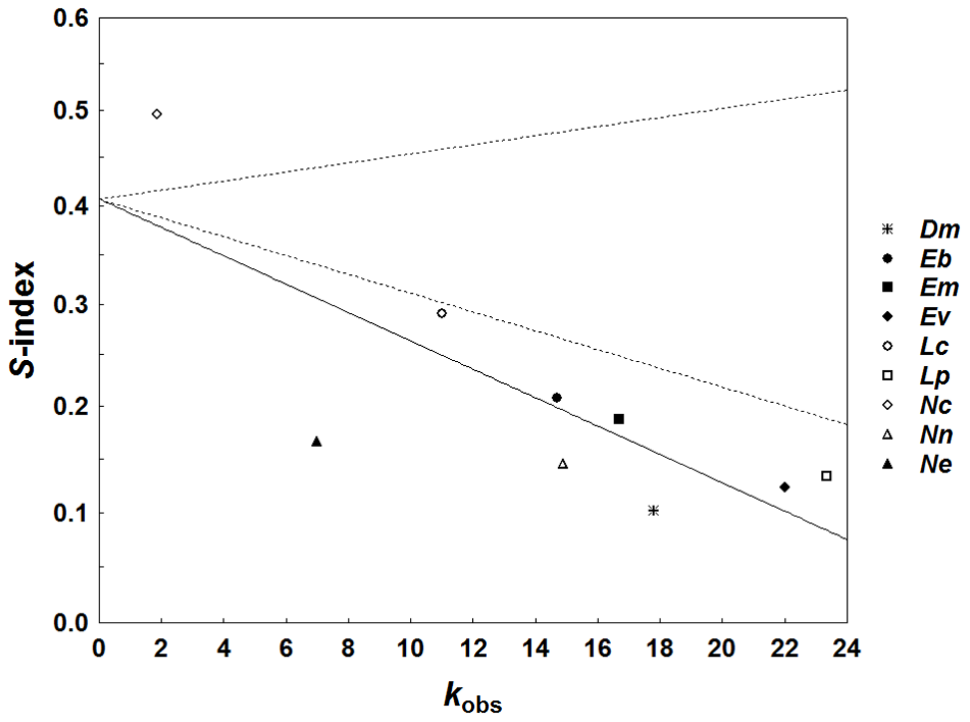
Association of the effective-number-index of paternity skew (*S*-index) and mean observed paternity frequencies (*k*_obs_) across the nine army ant species (represented by different symbols) for which *S* was estimated. The solid line indicates the slope of a phylogenetically corrected GLS regression (*b* = –0.005, *R* ^2^ = 0.63, *p* = 0.003) and dashed lines the 95% confidence interval of expected slopes with the same intercept under random paternity distribution. These were estimated from 100 Monte Carlo iterations of the GLS regression with randomly generated *S*-values (see text and Jaffé et al. [47] for details). An observed slope below the confidence limits is significantly steeper than expected from random.

Among all species of the first data set the GLS model, corrected for phylogeny [7], [65], [66], revealed a significantly negative slope (*b*) for a simple regression of paternity skew *B* on *k*_obs_ (likelihood ratio test with *df* = 1; *b* = –0.001, *R* ^2^ = 0.74, *χ* ^2^ = 7.12, *p* = 0.008; Figure 2). This also held true when using the sample size corrected *k*_est_ as predictor of *B* (*b* = –0.001, *R* ^2^ = 0.73, *χ* ^2^ = 7.10, *p* = 0.008). Likewise, for the second data set the observed slope of *S* on *k*_obs_ was significantly negative (*b* = –0.005, *R* ^2^ = 0.63, *χ* ^2^ = 8.92, *p* = 0.003; Figure 3) and steeper than randomly expected slopes with a 95% confidence interval ranging from –0.003 to 0.002. Simple GLS regressions on colony size were not significant, neither of paternity frequency, nor of paternity skew among all nine species (*k*_obs_: *b* = 0.712, *R* ^2^ = 0.00003, *χ* ^2^ = 0.01, *p* = 0.90; *S*-index: *b* = –0.015, *R* ^2^ = 0.03, *χ* ^2^ = 0.27, *p* = 0.61; Figures S1 and S2).

**Figure 4:**
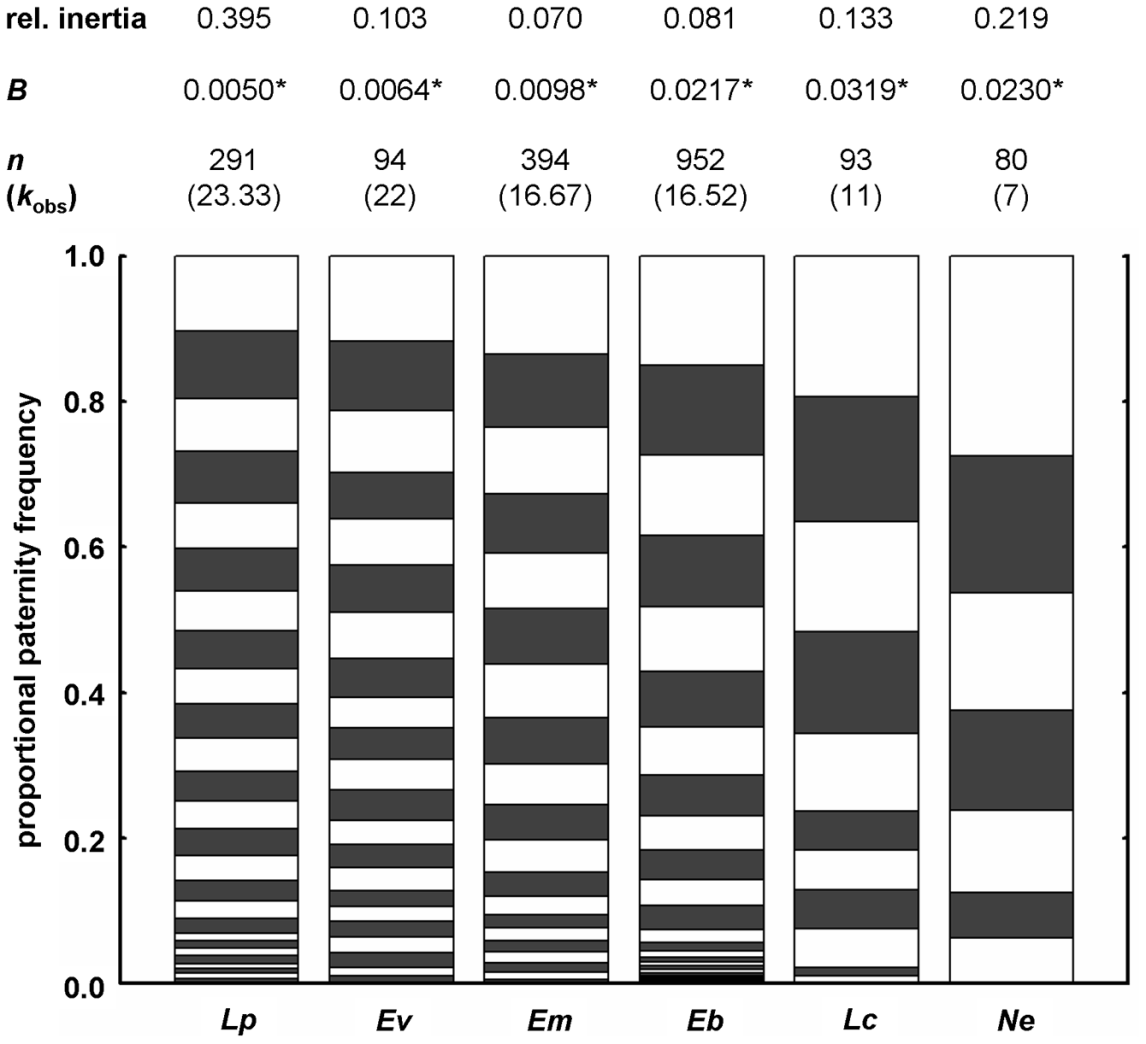
Paternity distribution of six Neotropical army ants (1–6 colonies) ranked according to mean observed paternity frequencies (*k*_obs_). Alternately shaded bars show the proportional paternity of all patrilines (summed over colonies for each species). Mean *k*_obs_, total number of assigned workers (*n*), paternity skew (*B*-index with asterisks marking significance per species) and relative inertia of the correspondence analysis are shown above the bars. The overall *χ* ^2^-statistics of the correspondence analysis indicates significant deviation from homogeneity of the paternity distribution among species (*χ* ^2^ = 198.67, *df* = 125, *p*<0.001). Relative inertia give the proportional contribution of each species to this deviation [67], showing the highest values for the species with the highest and lowest *k*_obs_ (*L. praedator* and *N. esenbeckii*, respectively).

The correspondence analysis showed significant deviation from homogeneity in the paternity share of patrilines among the six species of the first data set (overall *χ* ^2^ = 198.67, *df* = 125, *p*<0.001; Figure 4). Those species with the highest and lowest paternity frequencies (*L. praedator* and *N. esenbeckii*) contributed most to this differentiation in paternity skew, indicated by the highest relative inertia (0.395 and 0.219; Figure 4). Absolute patriline frequencies were summed over colonies per species for this analysis. Within species differences in paternity skew among colonies were not significant (Figure S3).

## Discussion

### Paternity frequencies

Effective paternity frequencies were high (up to 25.51) in all five army ant species analyzed in this study (Table 1), supporting the idea of ancestral evolution of extreme polyandry in army ants in close association with the army ant adaptive syndrome [7], [16]. Thus, all so far analyzed species exceed (most of them by far) the threshold above which a reduction of intracolonial genetic relatedness with further matings becomes negligible [27]–[29]. At this level of extreme polyandry fitness benefits resulting from genotypic variance can nevertheless be gained at colony level from mutualistic benefits of diverse, interacting genotypes that are more than the sum of their parts (social heterosis, [30]). Likewise, the maintenance of rare specialized genotypes could be advantageous at colony level [29]. For instance *L. praedator*, with the highest paternity frequencies and up to nine patrilines that are represented by only one worker (Table S3), is supportive to the latter hypothesis.

The only known not highly polyandrous army ant *N. carolinensis* does not contradict these hypotheses because in this species polyandry is secondarily replaced by polygyny with up to 19 queens [39], [40]. It has been argued that polyandry and polygyny can be alternative, negatively correlated strategies to increase intracolonial variance in social insects [17]–[19], [69].

### Population structure and relatedness

Colony fission in combination with male-biased sex ratios [4], [6], [9] predisposes army ants for small effective population sizes (*N*_e_), which severely increases the risk of genetic depletion and inbreeding [11], [13], [15]. Polyandry has been suggested as an escape route from these negative effects [11], [36] and indeed evidence for inbreeding is weak in army ants [16], [35], [36]. Male dispersal markedly facilitates gene flow [35], [37], and queens of *E. burchellii* can boost *N*_e_ by preferentially mating with unrelated males [36]. Also in our study we found only weak population sub-structuring (*F*_ST_ 0.019 and 0.069 for *E. mexicanum* and *L. praedator*) with high levels of heterozygosity (*H*_S_ 0.70 and 0.80, respectively), indicating high genetic variability and gene flow in both species. Some slight sub-structuring can nevertheless be expected because in army ants not all colonies of a population produce males in a given season [4], so that queens may have mated with different male population in different seasons.

Slightly positive and negative mean inbreeding coefficients (*F*_IS_ 0.039 and –0.016) in *E. mexicanum* and *L. praedator*, respectively, ranged well within previously observed values in other army ants (Figure 1). Negative *F*_IS_ as a result of heterozygote excess has also been found in *E. burchellii* [35] and, in combination with small *N*_e_, can be a signal of outbreeding [70]–[73], e.g., if queens preferentially mate with males from distinct sub-populations. Slightly positive *F*_IS_, as previously also found in *N. nigrescens* [16], however, not necessarily results from sib-mating, but may reflect slight heterozygote deficiency due to the observed population sub-structuring or due to the relatedness among some workers, from which *H*_O_ was estimated [15].

The average regression genetic relatedness among workers in *E. mexicanum* and *L. praedator* did not differ from the expected pedigree relatedness over all colonies (Table 2), confirming the absence of inbreeding, which was further supported by the lack of any relatedness between queens and their mates (in *E. mexicanum* even with a negative tendency). This and the significantly lower relatedness among workers compared to pedigree relatedness in colony *Em*2 (Table 2) again suggests outbreeding, as previously observed in *E. burchellii* [36]. In *L. praedator* a slightly positive average relatedness among males may reflect an uneven contribution of the surrounding colonies to the queens’ sires [35], [36]. This bias can explain the observed linkage between some loci pairs in *L. praedator*, but seems too small to significantly increase relatedness among workers.

Thus, our data illustrates the capability of army ant queens to avoid inbreeding by multiple mating with males to which they are unrelated to. At population level this seems to efficiently overcome the *N*_e_ constraints of the army ant syndrome by maintaining gene flow and genetic diversity. E.g., Chapman and Bourke [12] showed that the estimated *N*_e_ of an island population of *E. burchellii* increased from 75, assuming monandry, to 112 if assuming extreme polyandry. In contrast, in monandrous social insects small *N*_e_ often leads to high population sub-structuring and low heterozygosity ([74] for bumblebees, [75] for ants, and references therein).

### Paternity skew and colony size

A strong paternity skew can diminish the effect of multiple mating by shifting the average intracolonial relatedness towards that of effective monandry [19], [34], [44]–[47]. According to the genetic variance hypothesis for the evolution of polyandry [17]–[22] a high intracolonial genotypic variance is an important parameter for colony fitness. Hence, it would be beneficial for queens to equalize the paternity share among siring males. Indeed, our data show that paternity skews decrease with an increasing mean number of sires of a queen (observed and sample size corrected paternity frequencies). In fact, the two colonies *Lp*1 and *Lp*3 (*L. praedator*) with the highest observed paternity frequencies (*k*_obs_ = 26) were also the ones with *B*-indices indifferent from a random paternity skew in our first data set (Table 1). Thus, this study provides evidence that intracolonial genotypic variance is an important factor for the evolution of polyandry, even in its most extreme form. Moreover, it is to our knowledge the first to show a negative association between paternity skew and paternity frequency across species within a clade of extremely polyandrous social insects, confirming earlier findings across social Hymenoptera in general [47].

Such a negative association can be interpreted as a trade-off across species between high genotypic variance and high relatedness within colonies. According to Hamilton’s inclusive fitness concept [76], the latter is an important prerequisite for social cohesion [19], [22], [28], [34], [47], [69]. In the case of extreme polyandry natural selection may favor queens to maximize intracolonial genotypic variance by paternity equalization, since the loss of relatedness with additional matings becomes negligible anyway. In contrast, in polyandrous insects with relatively low paternity frequencies, competition among males often leads to precedence of the first or last sire and a strong paternity skew [46], [77]–[80].

According to our phylogenetically corrected data, males appear to have little power to influence paternity skew in army ants, whereas queens seem to be adapted to equalize paternity. The correspondence analysis confirms significant paternity skew differences among the six species of the first data set, with peaks at the most extreme paternity frequencies (Figure 4), but not among colonies within species (Figure S3). Our findings fit the data on honeybees (genus *Apis*), the only other social Hymenoptera matching army ants in their high degree of polyandry [24], [27], [32], [52]. Honeybees also exhibit similar degrees of paternity skew and provide evidence that queens indeed control paternity equalization [68], [77]. Further evidence for queen control over sperm use by means of cryptic female choice [81] comes from polyandrous leaf-cutter ants [82], [83].

If queens mate multiply for sperm limitation reasons [31]–[34] using all available sperm, large colonies should exhibit skewed paternities, since males differ naturally in sperm number and contribution [27], [32], [84]. Moreover, under the sperm limitation hypothesis paternity frequencies are expected to increase with colony size. However, colony size was not significantly associated with *k*_obs_ (Figure S1), and showed a trend of a negative association (though not significant) with the *S*-index of paternity skew over nine army ant species (Figure S2). Thus, for army ants, this study neither supports nor rejects the sperm limitation hypothesis as an additional factor for the evolution of extreme polyandry.

### Conclusion

Based on our results obtained from the analysis of paternity skew and genetic colony structure of nine army ants species, we suggest two main drivers for the evolution of extreme polyandry in army ants and in social insects in general. First, by mating with many unrelated males, queens seem to counteract problems connected with limited *N*_e_ [11]–[13], [36], facilitating gene flow and reducing the risk of inbreeding. This is supported by the fact that high polyandry in ants and honeybees is often associated with colony fission [16], [28], [84], which particularly reduces *N*_e_ (but see [85], [86]).

Second, in concordance with the genetic variance hypothesis polyandry may increase colony efficiency and parasite resistance [17]–[26], even beyond the expected threshold of six to ten matings [27]–[29], which can plausibly be explained by the principle of social heterosis [30]. Thus, an adaptive value of extreme polyandry, as suggested by Kraus et al. [87], seems more likely than selective neutrality, as proposed by Kronauer et al. [16], especially because the costs of mating are probably never zero. Even for army ant queens, which mate within the protection of the colony [4], [6] (reducing the risk of predation), potential costs such as energetic loss or disease/parasite transmission [17], [29] remain. The sperm limitation hypothesis [31]–[34] cannot be excluded here as an additional driver of extreme polyandry, but seems to play a minor role in army ants, fitting recent findings in stingless bees which can have enormously large colonies even in spite of monandrous queens [85].

Given our results, a possible scenario for the evolution of extreme polyandry in army ants could be that increasingly efficient group predation [1], [4]–[6], [9], [10] made colony fission as mode of reproduction obligate (requirement of a minimal worker number), which increased *N*_e_ limitations and led selection to favor higher paternity frequencies. The resulting genotypic variance, in turn, might have further boosted colony efficiency finally leading to the highly specialized life history of the army ant adaptive syndrome [6]–[8].

## Supporting Information

Figure S1 **Association of mean observed paternity frequencies (** ***k*** **_obs_) and average approximate colony sizes across the nine army ant species (represented by different symbols) for which also paternity skew was analyzed.** The solid line indicates the slope of a phylogenetically corrected GLS regression (*b* = 0.712, *R* ^2^ = 0.00003, *p* = 0.90) and dashed lines the 95% confidence interval.
(TIF)

Figure S2 **Association of the effective-number-index of paternity skew (** ***S*** **-index) and average approximate colony sizes across nine army ant species.** The solid line indicates the slope of a phylogenetically corrected GLS regression (*b* = –0.015, *R* ^2^ = 0.03, *p* = 0.61) and dashed lines the 95% confidence interval.
(TIF)

Figure S3 **Paternity distribution among 3–6 colonies of the three Neotropical army ant species** ***E. burchellii*** **(** ***Eb*** **),** ***E. mexicanum*** **(** ***Em*** **) and** ***L. praedator*** **(** ***Lp*** **).** Alternately shaded bars show the proportional paternity of all patrilines. *k*_obs_, number of assigned workers (*n*), paternity skew (*B*-index with asterisks marking Bonferroni adjusted significance) and relative inertia of the correspondence analysis are shown above the bars for each colony. Relative inertia give the proportional contribution of each colony to deviation from homogeneity of the paternity distribution within a species. The *χ* ^2^-statistics of the correspondence analysis, given per species above the diagram, indicates no significant deviation among colonies for neither species.
(TIF)

Table S1 **Characteristics of five new microsatellite loci developed for the Neotropical army ant** ***Labidus praedator*** **.**
(DOC)

Table S2 **Cross-amplification data of 14 microsatellite loci among five Neotropical army ant species.**
(DOC)

Table S3 **Inferred queen and patriline genotypes of five Neotropical army ants.**
(DOC)

Protocol S1 **Microsatellite isolation protocol for** ***Labidus praedator*** **and cross-amplification tests.**
(DOC)
