# Supplementary figures and images for: The Evolution of Extreme Polyandry in Social Insects: Insights from Army Ants
Source: PLoS One. 2014 Aug 21;9(8):e105621. doi: 10.1371/journal.pone.0105621 (PMC4140799; doi:10.1371/journal.pone.0105621)

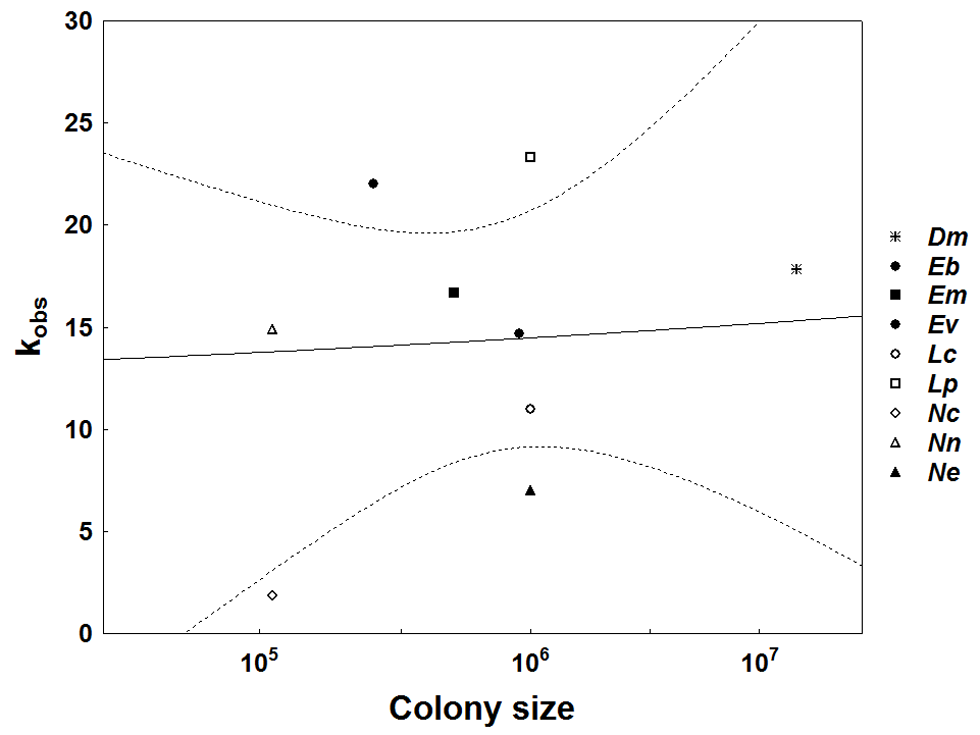

Supplement: Figure S1 — Association of mean observed paternity frequencies ( k obs) and average approximate colony sizes across the nine army ant species (represented by different symbols) for which also paternity skew was analyzed. The solid line indicates the slope of a phylogenetically corrected GLS regression (b = 0.712, R 2 = 0.00003, p = 0.90) and dashed lines the 95% confidence interval. (TIF) [file pone.0105621.s001.tif]

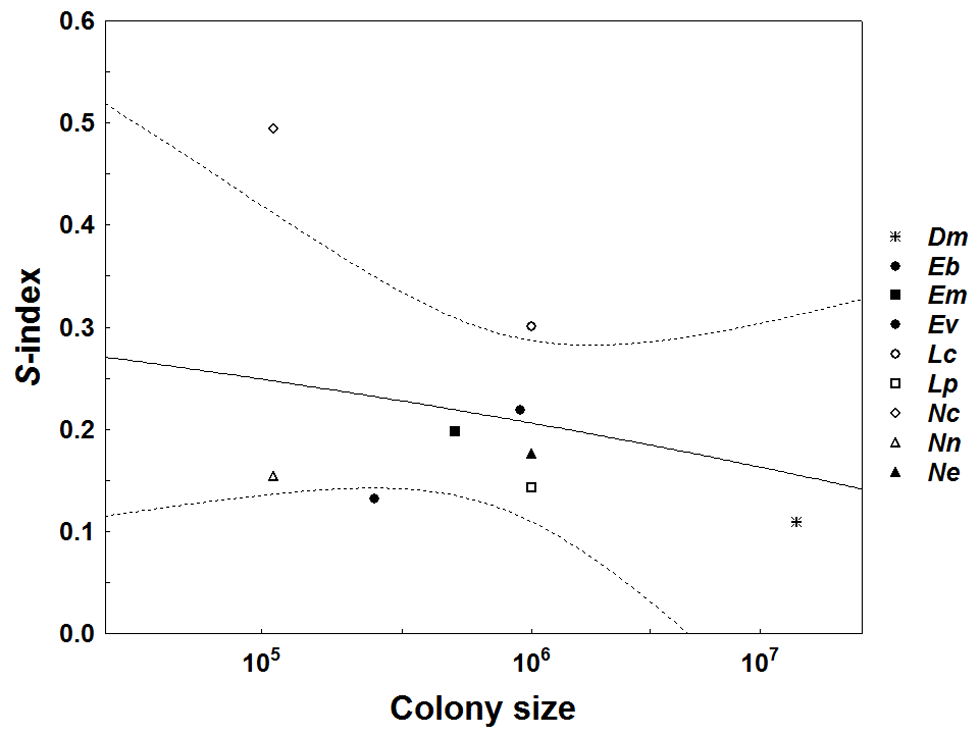

Supplement: Figure S2 — Association of the effective-number-index of paternity skew ( S -index) and average approximate colony sizes across nine army ant species. The solid line indicates the slope of a phylogenetically corrected GLS regression (b = –0.015, R 2 = 0.03, p = 0.61) and dashed lines the 95% confidence interval. (TIF) [file pone.0105621.s002.tif]

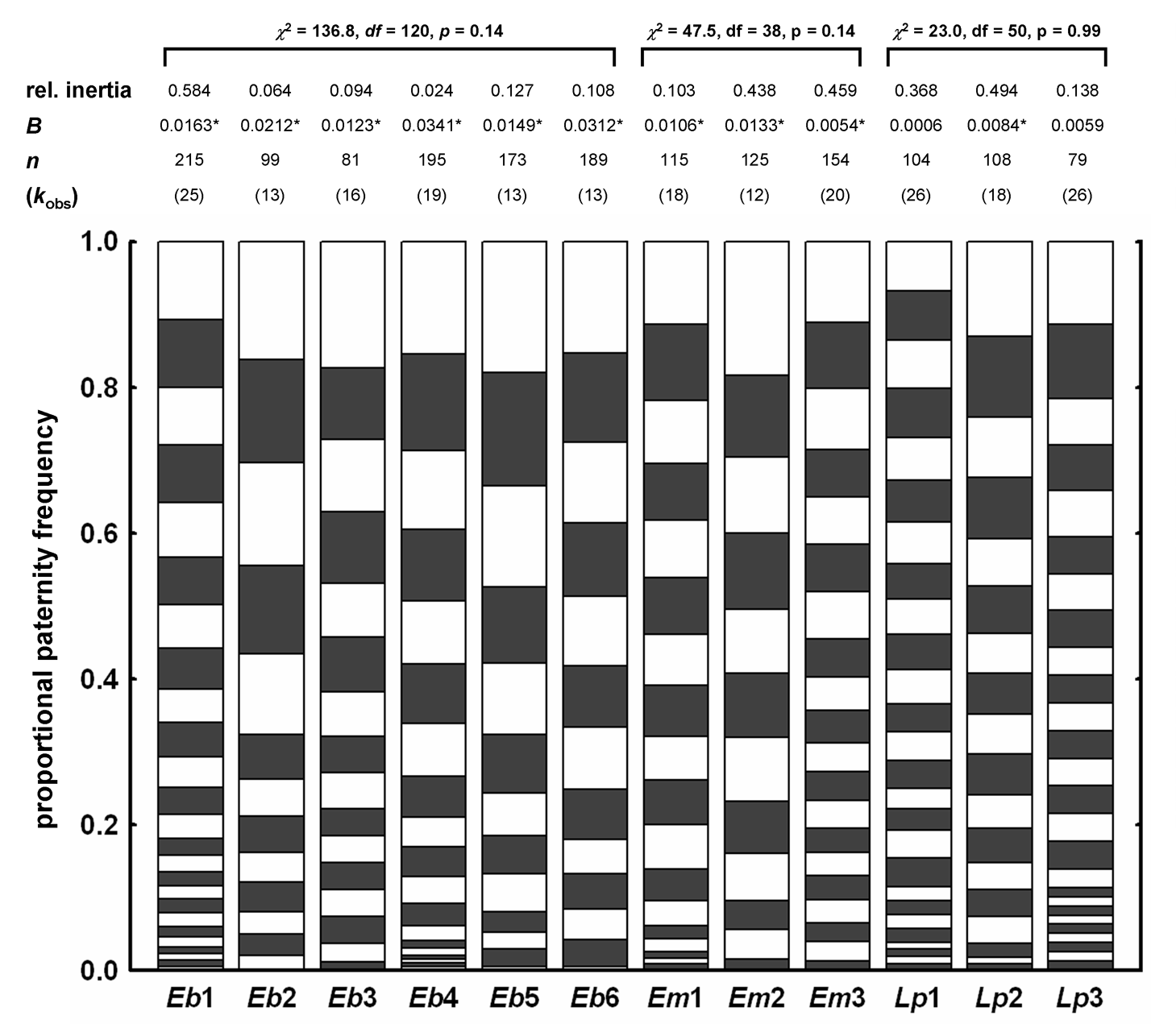

Supplement: Figure S3 — Paternity distribution among 3–6 colonies of the three Neotropical army ant species E. burchellii ( Eb ), E. mexicanum ( Em ) and L. praedator ( Lp ). Alternately shaded bars show the proportional paternity of all patrilines. k obs, number of assigned workers (n), paternity skew (B-index with asterisks marking Bonferroni adjusted significance) and relative inertia of the correspondence analysis are shown above the bars for each colony. Relative inertia give the proportional contribution of each colony to deviation from homogeneity of the paternity distribution within a species. The χ 2-statistics of the correspondence analysis, given per species above the diagram, indicates no significant deviation among colonies for neither species. (TIF) [file pone.0105621.s003.tif]
