# Supplementary material for: The Evolution of Extreme Polyandry in Social Insects: Insights from Army Ants
Source: PLoS One. 2014 Aug 21;9(8):e105621. doi: 10.1371/journal.pone.0105621 (PMC4140799; doi:10.1371/journal.pone.0105621)
Supplement: Table S1 — Characteristics of five new microsatellite loci developed for the Neotropical army ant Labidus praedator . (DOC) [file pone.0105621.s004.doc]

**Table S1.** Characteristics of five new microsatellite loci developed for the Neotropical army ant *Labidus praedator*.

| Locus | Primer sequence (5’-3’) | Repeat motif | Size  range (bp) | *n* | *A* | *H*_O_ | *H*_S_ | GenBank accession no. |
| --- | --- | --- | --- | --- | --- | --- | --- | --- |
| Lp2 | F: GGACCAACATTGAAATCGAAATA | (AG)_18_AA(AG)_8_ | 115–127 | 282 | 6 | 0.78 | 0.79 | KF969232 |
|  | R: CGGATTTGGGAAGAAAACCTAT |  |  |  |  |  |  |  |
| Lp4 | F: GCTTCAATTGACAGATAACGCTAA | (AG)_19_ | 84–102 | 289 | 10 | 0.89 | 0.82 | KF969233 |
|  | R: AGGTCGAATTTATGCGTAGTTGC |  |  |  |  |  |  |  |
| Lp14a | F: GCGCGCCTTACATTCTCC | (CT)_11_ | 154–170 | 291 | 6 | 0.75 | 0.75 | KF969234 |
|  | R: CGTCTTGATAGCTCCTTCTCGTT |  |  |  |  |  |  |  |
| Lp30 | F: TCGAATTTATGTCGGAATCAAAC | (GA)_31_ | 314–339 | 276 | 11 | 0.86 | 0.84 | KF969235 |
|  | R: CGTCCATTATCCCTAATATCGTTG |  |  |  |  |  |  |  |
| Lp38 | F: AATTAAGCGATCGTAGGATTATGC | (GA)_20_AA(GA)_2_ | 126–172 | 291 | 13 | 0.92 | 0.87 | KF969236 |
|  | R: ACCTGCAGTAAATACGTTGGTAAT |  |  |  |  |  |  |  |

Annealing temperatures were 54°C for all loci. *n*, number of screened workers (from three colonies); *A*, observed number of alleles; *H*_O_ and *H*_S_, observed and expected heterozygosity.
