# Supplementary material for: The Evolution of Extreme Polyandry in Social Insects: Insights from Army Ants
Source: PLoS One. 2014 Aug 21;9(8):e105621. doi: 10.1371/journal.pone.0105621 (PMC4140799; doi:10.1371/journal.pone.0105621)
Supplement: Table S2 — Cross-amplification data of 14 microsatellite loci among five Neotropical army ant species. (DOC) [file pone.0105621.s005.doc]

**Table S2.** Cross-amplification data of 14 microsatellite loci among five Neotropical army ant species.

| **Species (colonies)** | **Eb04** | **Eb10** | **Eb14** | **Eb21** | **Eb24** | **Eb25** | **Eb42** | **Eb51** | **DmoD** | **Lp2** | **Lp4** | **Lp14a** | **Lp30** | **Lp38** |
| --- | --- | --- | --- | --- | --- | --- | --- | --- | --- | --- | --- | --- | --- | --- |
| *E. mexicanum* (3) | ++ | ++ | + | ++ | ++ | ++ | ++ | ++ | ++ | + | ++ | +0 | - | ++ |
| *E. vagans* (1) | ++ | ++ | ++ | ++ | - | ++ | ++ | ++ | ++ | ++ | ++ | - | - | ++ |
| *L. coecus* (1) | - | ++ | - | - | + | ++ | - | + | + | + | - | ++ | - | - |
| *L. praedator* (3) | ++ | - | - | - | + | ++ | ++ | - | ++ | ++ | ++ | ++ | ++ | ++ |
| *N. esbecki* (2) | - | - | - | - | + | ++ | - | ++ | - | ++ | ++ | ++ | - | ++ |

Six individuals from one to three colonies were tested. The five Lp loci were newly developed for *Labidus praedator* in this study (see Protocol S1 and Table S1), eight Eb loci have been developed for *Eciton burchellii* [1], and DmoD for *Dorylus molestus* [2]. ++, polymorphic amplification; +0, polymorphic amplification but evidence for null alleles; +, monomorphic amplification; -, no amplification.

**References**

1. Denny AJ, Franks NR, Edwards KJ (2004) Eight highly polymorphic microsatellite markers for the army ant *Eciton burchellii*. Mol Ecol Notes 4: 234–236. doi:10.1111/j.1471-8286.2004.00627.x.

2. Kronauer DJC, Boomsma JJ, Gadau J (2004) Microsatellite markers for the driver ant *Dorylus* (*Anomma*) *molestus*. Mol Ecol Notes 4: 289–290. doi:10.1111/j.1471-8286.2004.00645.x.
