# Supplementary material for: The Evolution of Extreme Polyandry in Social Insects: Insights from Army Ants
Source: PLoS One. 2014 Aug 21;9(8):e105621. doi: 10.1371/journal.pone.0105621 (PMC4140799; doi:10.1371/journal.pone.0105621)
Supplement: Table S3 — Inferred queen and patriline genotypes of five Neotropical army ants. (DOC) [file pone.0105621.s006.doc]

Table S3. Inferred queen and patriline genotypes (allele lengths of all polymorphic loci are given in base pairs) of the five Neotropical army ants *E. mexicanum*, *E. vagans*, *L. coecus*, *L. praedator* and *N. esenbeckii*. These genotypes were used to estimate the population wide allele frequencies, expected heterozygosities (*H*_S_) and population structure (see main text).

*p*, non-detection error of patrilines per colony, i.e. the probability to obtain two males with the same allele combination at all loci

*n*, number of assigned workers per patriline

*Q*, the male allele could not be unambiguously determined because workers shared the same two alleles as the heterozygote queen. In this case the particular locus was entered as unknown in the analyses.

-, actually unknown locus due to poor amplification

| **Colony** | ***Em*1** | **(*p* = 1.46 × 10^–5^)** | |  |  |  |  |  |  |  |  |
| --- | --- | --- | --- | --- | --- | --- | --- | --- | --- | --- | --- |
|  |  |  |  |  |  |  |  |  |  |  |  |
| **Locus** | **Eb04** | **Eb10** | **Eb21** | **Eb24** | **Eb25** | **Eb42** | **Eb51** | **DmoD** | **Lp4** | **Lp38** |  |
|  |  |  |  |  |  |  |  |  |  |  |  |
| **Queen** | 102 | 140 | 128 | 120 | 139 | 124 | 131 | 152 | 82 | 118 |  |
|  | 104 | 152 | 128 | 122 | 139 | 128 | 137 | 166 | 84 | 132 |  |
|  |  |  |  |  |  |  |  |  |  |  |  |
| **Patriline (*n*)** |  |  |  |  |  |  |  |  |  |  |  |
| **1 (13)** | 92 | 152 | 128 | 122 | 160 | 122 | 139 | 164 | 70 | 132 |  |
| **2 (12)** | 92 | 154 | 128 | 120 | 139 | 82 | 137 | 150 | 74 | 132 |  |
| **3 (10)** | 96 | 148 | 151 | 120 | 139 | 122 | 135 | 164 | 84 | 132 |  |
| **4 (9)** | 92 | 140 | 128 | 122 | 139 | 122 | 135 | 168 | 82 | 132 |  |
| **5 (9)** | 92 | 148 | 128 | 120 | 139 | 128 | 135 | 166 | 70 | 132 |  |
| **6 (9)** | 96 | 140 | 128 | 122 | 139 | 130 | 137 | 162 | 70 | 120 |  |
| **7 (8)** | 96 | 140 | 130 | 120 | 139 | 132 | 133 | 162 | 70 | 132 |  |
| **8 (8)** | 96 | 154 | 128 | 120 | 160 | 126 | 133 | 164 | 74 | 132 |  |
| **9 (7)** | 96 | 148 | 128 | 120 | 139 | 128 | 137 | 147 | 82 | 132 |  |
| **10 (7)** | 96 | 148 | 128 | 120 | 160 | 128 | 139 | 164 | 74 | 118 |  |
| **11 (7)** | 96 | 152 | 128 | 120 | 139 | 130 | 137 | 158 | 74 | 134 |  |
| **12 (5)** | 92 | 140 | 149 | 122 | 156 | 128 | 141 | 164 | 74 | 118 |  |
| **13 (4)** | 96 | 148 | 128 | 126 | 156 | 126 | 141 | 166 | 74 | 118 |  |
| **14 (2)** | 96 | 148 | 128 | 120 | 160 | 126 | 133 | 168 | 74 | 132 |  |
| **15 (2)** | 96 | 154 | 128 | 120 | 139 | 122 | 133 | 164 | 70 | 118 |  |
| **16 (1)** | 92 | *Q* | 128 | 122 | 139 | 82 | 137 | 162 | 86 | *Q* |  |
| **17 (1)** | 96 | 140 | 128 | 124 | 139 | 122 | 133 | 158 | 70 | 134 |  |
| **18 (1)** | 96 | 148 | 151 | *Q* | 156 | 126 | 117 | 162 | 70 | 132 |  |
|  |  |  |  |  |  |  |  |  |  |  |  |
| **Colony** | ***Em*2** | **(*p* = 6.74 × 10^–6^)** | |  |  |  |  |  |  |  |  |
|  |  |  |  |  |  |  |  |  |  |  |  |
| **Queen** | 96 | 140 | 128 | 120 | 139 | 130 | 137 | 150 | 70 | 118 |  |
|  | 102 | 148 | 128 | 144 | 158 | 134 | 141 | 162 | 82 | 132 |  |
|  |  |  |  |  |  |  |  |  |  |  |  |
| **Patriline (*n*)** |  |  |  |  |  |  |  |  |  |  |  |
| **1 (23)** | 92 | 140 | 128 | 120 | 139 | 130 | 151 | 166 | 82 | 134 |  |
| **2 (14)** | 98 | 148 | 128 | 120 | 156 | 130 | 141 | 158 | 82 | 134 |  |
| **3 (13)** | 92 | 140 | 128 | 120 | 139 | 82 | 143 | 168 | 70 | 134 |  |
| **4 (13)** | 92 | 140 | 149 | 120 | 139 | 128 | 137 | 158 | 82 | 120 |  |
| **5 (11)** | 92 | 154 | 161 | 118 | 158 | 132 | 141 | 166 | 74 | 134 |  |
| **6 (11)** | 96 | 148 | 128 | 144 | 139 | 130 | 133 | 160 | 84 | 118 |  |
| **7 (11)** | 98 | 140 | 141 | 120 | 139 | 130 | 119 | 164 | 82 | 134 |  |
| **8 (9)** | 98 | 148 | 139 | 148 | 160 | 122 | 141 | 166 | 70 | 132 |  |
| **9 (8)** | 98 | 140 | 128 | 120 | 139 | 132 | 137 | 168 | 70 | 132 |  |
| **10 (5)** | 92 | 140 | 128 | 116 | 139 | 82 | 143 | 168 | 86 | 136 |  |
| **11 (5)** | 96 | 152 | 149 | 120 | 139 | 130 | 139 | 162 | 74 | 132 |  |
| **12 (2)** | *Q* | 154 | 161 | 118 | *Q* | *Q* | 141 | 164 | 82 | 134 |  |
|  |  |  |  |  |  |  |  |  |  |  |  |
| **Colony** | ***Em*3** | **(*p* = 1.87 × 10^–5^)** | |  |  |  |  |  |  |  |  |
|  |  |  |  |  |  |  |  |  |  |  |  |
| **Queen** | 98 | 140 | 128 | 116 | 139 | 128 | 137 | 166 | 74 | 118 |  |
|  | 100 | 140 | 128 | 120 | 139 | 130 | 141 | 168 | 82 | 120 |  |
|  |  |  |  |  |  |  |  |  |  |  |  |
| **Patriline (*n*)** |  |  |  |  |  |  |  |  |  |  |  |
| **1 (17)** | 96 | 148 | 128 | 120 | 139 | 132 | 135 | 166 | 82 | 136 |  |
| **2 (14)** | 98 | 152 | 130 | 120 | 139 | 128 | 135 | 164 | 84 | 132 |  |
| **3 (13)** | 96 | 140 | 149 | 120 | 139 | 82 | 91 | 174 | 74 | 136 |  |
| **4 (10)** | 96 | 148 | 128 | 122 | 139 | 132 | 135 | 176 | 82 | 132 |  |
| **5 (10)** | 96 | 152 | 130 | 120 | 139 | 130 | 109 | 168 | 82 | 118 |  |
| **6 (10)** | 96 | 154 | 128 | 120 | 139 | 130 | 139 | 152 | 74 | 134 |  |
| **7 (10)** | 100 | 140 | 128 | 120 | 139 | 126 | 137 | 166 | 74 | 132 |  |
| **8 (8)** | 92 | 140 | 128 | 120 | 158 | 130 | 137 | 164 | 86 | 118 |  |
| **9 (7)** | 92 | 148 | 128 | 120 | 139 | 130 | 133 | 168 | 74 | 132 |  |
| **10 (7)** | 100 | 152 | 128 | 120 | 139 | 126 | 135 | 150 | 74 | 132 |  |
| **11 (6)** | 92 | 148 | 128 | 122 | 139 | 132 | 135 | 166 | 82 | 132 |  |
| **12 (6)** | 96 | 140 | 128 | 120 | 139 | 82 | 135 | *Q* | 74 | 132 |  |
| **13 (6)** | 96 | 148 | 128 | 122 | 158 | 130 | 141 | 166 | 82 | 134 |  |
| **14 (5)** | 92 | 148 | 130 | 120 | 139 | 128 | 139 | 150 | 74 | 132 |  |
| **15 (5)** | 92 | 152 | 128 | 122 | 156 | 130 | 141 | 166 | 82 | 134 |  |
| **16 (5)** | 96 | 140 | 128 | 118 | 139 | 130 | 91 | 166 | 74 | 136 |  |
| **17 (5)** | 96 | 152 | 128 | 122 | 156 | 130 | 137 | 150 | *Q* | 134 |  |
| **18 (4)** | 92 | 152 | 160 | 116 | 158 | 130 | 137 | 166 | 74 | 134 |  |
| **19 (4)** | 100 | 140 | 149 | 120 | 139 | 126 | 137 | 164 | 74 | 132 |  |
| **20 (2)** | 96 | 148 | 149 | *Q* | 160 | 120 | 137 | 162 | 70 | 132 |  |

| **Colony** | ***Ev*1** | **(*p* = 2.21 × 10^–9^)** | |  |  |  |  |  |  |  |  |
| --- | --- | --- | --- | --- | --- | --- | --- | --- | --- | --- | --- |
|  |  |  |  |  |  |  |  |  |  |  |  |
| **Locus** | **Eb04** | **Eb10** | **Eb14** | **Eb21** | **Eb25** | **Eb42** | **Eb51** | **DmoD** | **Lp2** | **Lp4** | **Lp38** |
|  |  |  |  |  |  |  |  |  |  |  |  |
| **Queen** | 127 | 158 | 156 | 148 | 146 | 106 | 150 | 166 | 116 | 98 | 130 |
|  | 137 | 172 | 156 | 182 | 148 | 106 | 156 | 178 | 128 | 98 | 136 |
|  |  |  |  |  |  |  |  |  |  |  |  |
| **Patriline (*n*)** |  |  |  |  |  |  |  |  |  |  |  |
| **1 (11)** | 137 | 166 | 156 | 156 | 144 | 88 | 162 | 178 | 114 | 96 | 146 |
| **2 (9)** | 123 | 182 | 164 | 168 | 148 | 96 | 158 | 174 | 118 | 98 | 134 |
| **3 (8)** | 125 | 162 | 154 | 140 | 136 | 88 | 154 | 168 | 118 | 100 | 130 |
| **4 (6)** | 117 | 164 | 154 | 140 | 144 | 108 | 140 | 138 | 118 | 100 | 128 |
| **5 (6)** | 125 | 170 | 158 | 162 | 144 | 88 | 132 | 160 | 128 | 100 | 128 |
| **6 (6)** | 127 | 160 | 164 | 170 | 148 | 108 | 152 | 162 | 116 | 100 | 142 |
| **7 (6)** | 145 | 158 | 154 | 140 | 148 | 90 | 148 | 168 | 118 | 96 | 128 |
| **8 (5)** | 127 | 166 | 154 | 140 | 140 | 106 | 134 | 178 | 118 | 98 | 122 |
| **9 (4)** | 111 | 192 | 154 | 164 | 144 | 90 | 148 | 184 | 120 | 96 | 122 |
| **10 (4)** | 131 | *Q* | 164 | 166 | 146 | 90 | 148 | 168 | 118 | 102 | 118 |
| **11 (4)** | 135 | 162 | 162 | 158 | 144 | 88 | 166 | - | 114 | 100 | 122 |
| **12 (4)** | 151 | 156 | 154 | 142 | 154 | 88 | 132 | 182 | 118 | 94 | 128 |
| **13 (3)** | 125 | 162 | 158 | 162 | 138 | 88 | 132 | - | 114 | 98 | 122 |
| **14 (3)** | 137 | 158 | 154 | 172 | 144 | 108 | 140 | 172 | 122 | 100 | 130 |
| **15 (3)** | 137 | *Q* | 154 | 140 | 144 | 88 | 140 | 138 | 118 | 100 | 128 |
| **16 (2)** | 111 | 156 | 154 | 146 | 136 | 88 | 140 | 170 | 118 | 98 | 140 |
| **17 (2)** | 133 | 156 | 166 | 168 | 144 | 92 | 142 | 176 | 118 | 100 | 142 |
| **18 (2)** | 139 | 156 | 156 | 168 | 146 | 114 | 160 | 166 | 118 | 100 | 120 |
| **19 (2)** | 141 | 172 | 164 | 166 | 148 | 116 | 142 | 170 | 114 | 96 | 122 |
| **20 (2)** | 147 | 156 | 156 | 164 | 150 | 112 | 134 | - | 116 | 96 | 130 |
| **21 (1)** | 135 | 186 | 154 | 148 | 146 | 88 | *Q* | 168 | 114 | 102 | 122 |
| **22 (1)** | 145 | 166 | 158 | 170 | 144 | 88 | 148 | 156 | 114 | 94 | 126 |

| **Colony** | ***Lc*1** | **(*p* = 2.32 × 10^–2^)** | |  |  |  |  |  |  |  |  |
| --- | --- | --- | --- | --- | --- | --- | --- | --- | --- | --- | --- |
|  |  |  |  |  |  |  |  |  |  |  |  |
| **Locus** | **Eb10** | **Eb25** | **Lp14a** |  |  |  |  |  |  |  |  |
|  |  |  |  |  |  |  |  |  |  |  |  |
| **Queen** | 138 | 142 | 146 |  |  |  |  |  |  |  |  |
|  | 138 | 144 | 150 |  |  |  |  |  |  |  |  |
|  |  |  |  |  |  |  |  |  |  |  |  |
| **Patriline (*n*)** |  |  |  |  |  |  |  |  |  |  |  |
| **1 (18)** | 138 | 146 | 138 |  |  |  |  |  |  |  |  |
| **2 (16)** | 136 | 162 | 152 |  |  |  |  |  |  |  |  |
| **3 (14)** | 138 | 162 | 148 |  |  |  |  |  |  |  |  |
| **4 (13)** | 138 | 144 | 142 |  |  |  |  |  |  |  |  |
| **5 (10)** | 136 | 144 | 148 |  |  |  |  |  |  |  |  |
| **6 (5)** | 138 | 148 | 148 |  |  |  |  |  |  |  |  |
| **7 (5)** | 138 | 146 | 144 |  |  |  |  |  |  |  |  |
| **8 (5)** | 138 | 142 | 150 |  |  |  |  |  |  |  |  |
| **9 (5)** | 138 | 142 | 144 |  |  |  |  |  |  |  |  |
| **10 (1)** | 138 | 146 | *Q* |  |  |  |  |  |  |  |  |
| **11 (1)** | 136 | 146 | 142 |  |  |  |  |  |  |  |  |

| **Colony** | ***Lp*1** | **(*p* = 1.67 × 10^–6^)** | |  |  |  |  |  |  |  |  |
| --- | --- | --- | --- | --- | --- | --- | --- | --- | --- | --- | --- |
|  |  |  |  |  |  |  |  |  |  |  |  |
| **Locus** | **Eb10** | **Eb25** | **Eb42** | **DmoD** | **Lp2** | **Lp4** | **Lp14a** | **Lp30** | **Lp38** |  |  |
|  |  |  |  |  |  |  |  |  |  |  |  |
| **Queen** | 194 | 164 | 116 | 172 | 117 | 88 | 166 | 331 | 138 |  |  |
|  | 218 | 164 | 136 | 172 | 123 | 88 | 168 | 335 | 138 |  |  |
|  |  |  |  |  |  |  |  |  |  |  |  |
| **Patriline (*n*)** |  |  |  |  |  |  |  |  |  |  |  |
| **1 (7)** | 188 | 166 | 149 | 174 | 119 | 92 | 170 | 333 | 136 |  |  |
| **2 (7)** | 212 | 164 | 114 | 182 | 119 | 96 | 166 | 325 | 132 |  |  |
| **3 (7)** | 216 | 164 | 94 | 184 | 127 | 100 | 154 | 327 | 136 |  |  |
| **4 (7)** | 226 | 164 | 141 | 176 | 123 | 90 | 166 | 327 | 132 |  |  |
| **5 (6)** | 214 | 168 | 151 | 174 | 119 | 92 | 164 | 331 | 130 |  |  |
| **6 (6)** | 216 | 164 | 116 | 172 | 123 | 96 | 154 | 331 | 134 |  |  |
| **7 (6)** | 230 | 164 | 145 | 184 | 119 | 92 | 166 | *Q* | 126 |  |  |
| **8 (5)** | 210 | 164 | 145 | 178 | 117 | 92 | 166 | 329 | 136 |  |  |
| **9 (5)** | 212 | 164 | 141 | 172 | 123 | 96 | 166 | 337 | 132 |  |  |
| **10 (5)** | 218 | 164 | 141 | 184 | 121 | 92 | 166 | 329 | 136 |  |  |
| **11 (5)** | 216 | 164 | 145 | 172 | 115 | 96 | 166 | 331 | 132 |  |  |
| **12 (4)** | 212 | 164 | 114 | 182 | 119 | 90 | 164 | 325 | 132 |  |  |
| **13 (4)** | 216 | 164 | 145 | 172 | 115 | 88 | *Q* | 331 | 134 |  |  |
| **14 (4)** | 216 | 164 | 145 | 172 | - | - | 154 | 331 | 134 |  |  |
| **15 (4)** | 228 | 164 | 94 | 182 | 117 | 88 | 166 | 329 | 142 |  |  |
| **16 (4)** | 228 | 164 | 116 | 176 | 119 | 90 | 168 | 333 | 138 |  |  |
| **17 (3)** | 216 | 160 | 141 | 184 | 117 | 86 | 166 | 339 | 156 |  |  |
| **18 (3)** | 228 | 160 | 141 | 176 | 117 | 90 | 166 | 331 | 128 |  |  |
| **19 (2)** | 196 | 164 | 141 | 182 | 119 | 90 | 164 | 325 | 172 |  |  |
| **20 (2)** | 216 | 164 | 139 | 176 | 123 | 88 | *Q* | 333 | 138 |  |  |
| **21 (2)** | 228 | 164 | 116 | 176 | 119 | 88 | *Q* | 339 | 138 |  |  |
| **22 (2)** | 228 | 164 | *Q* | 172 | 123 | 88 | *Q* | 333 | 132 |  |  |
| **23 (1)** | 196 | 164 | 141 | 182 | 119 | 90 | 162 | 325 | 172 |  |  |
| **24 (1)** | 210 | 160 | 172 | 174 | 121 | 92 | 168 | 339 | 142 |  |  |
| **25 (1)** | 228 | 164 | 139 | 172 | 123 | 88 | *Q* | 333 | 132 |  |  |
| **26 (1)** | *Q* | 164 | 116 | 176 | 119 | 90 | 168 | 333 | 132 |  |  |
|  |  |  |  |  |  |  |  |  |  |  |  |
| **Colony** | ***Lp*2** | **(*p* = 1.07 × 10^–6^)** | |  |  |  |  |  |  |  |  |
|  |  |  |  |  |  |  |  |  |  |  |  |
| **Queen** | 186 | 162 | 141 | 178 | 121 | 88 | 166 | 333 | 132 |  |  |
|  | 248 | 164 | 143 | 182 | 123 | 88 | 168 | 335 | 136 |  |  |
|  |  |  |  |  |  |  |  |  |  |  |  |
| **Patriline (*n*)** |  |  |  |  |  |  |  |  |  |  |  |
| **1 (14)** | 216 | 162 | 141 | 174 | 117 | 94 | 166 | 327 | 144 |  |  |
| **2 (12)** | 186 | 154 | 141 | 182 | 119 | 92 | 166 | 333 | 132 |  |  |
| **3 (9)** | 192 | 162 | 114 | 184 | 117 | 92 | 166 | 327 | 144 |  |  |
| **4 (9)** | 194 | 166 | 94 | 180 | 123 | 90 | 168 | 314 | 156 |  |  |
| **5 (7)** | 186 | 154 | 149 | 166 | 119 | 88 | 166 | 333 | 132 |  |  |
| **6 (7)** | 212 | 166 | 151 | 172 | 119 | 100 | 168 | 327 | 128 |  |  |
| **7 (6)** | 192 | 158 | 94 | 174 | 127 | 86 | 170 | 329 | 156 |  |  |
| **8 (6)** | 194 | 162 | 94 | 182 | 123 | 92 | 168 | 331 | 156 |  |  |
| **9 (6)** | 218 | 160 | 141 | 172 | 119 | 90 | 168 | 327 | 144 |  |  |
| **10 (6)** | 218 | 166 | 141 | 172 | 117 | 90 | 166 | 329 | 144 |  |  |
| **11 (5)** | 216 | 162 | 141 | 184 | 117 | 92 | 166 | 329 | 144 |  |  |
| **12 (5)** | 216 | 162 | 145 | 174 | 119 | 92 | 166 | 316 | 128 |  |  |
| **13 (4)** | 192 | - | 141 | 174 | 127 | 102 | 166 | 333 | 130 |  |  |
| **14 (4)** | 212 | - | 149 | 174 | 117 | 90 | 162 | 316 | 126 |  |  |
| **15 (4)** | 216 | 160 | 94 | 162 | 123 | 88 | 170 | 329 | 128 |  |  |
| **16 (2)** | 224 | 158 | 145 | 182 | 117 | 92 | 166 | 333 | 126 |  |  |
| **17 (1)** | - | 162 | 151 | 174 | 117 | 90 | 162 | 316 | 144 |  |  |
| **18 (1)** | - | 160 | 149 | 174 | 119 | 92 | 170 | 316 | 138 |  |  |
|  |  |  |  |  |  |  |  |  |  |  |  |
| **Colony** | ***Lp*3** | **(*p* = 7.91 × 10^–7^)** | |  |  |  |  |  |  |  |  |
|  |  |  |  |  |  |  |  |  |  |  |  |
| **Queen** | 192 | 164 | 114 | 172 | 117 | 98 | 164 | 327 | 126 |  |  |
|  | 210 | 164 | 116 | 184 | 127 | 102 | 166 | 329 | 154 |  |  |
|  |  |  |  |  |  |  |  |  |  |  |  |
| **Patriline (*n*)** |  |  |  |  |  |  |  |  |  |  |  |
| **1 (9)** | 192 | 164 | 130 | 172 | 123 | 86 | 168 | 318 | 140 |  |  |
| **2 (8)** | 216 | 164 | 94 | 180 | 127 | 100 | 162 | 329 | 140 |  |  |
| **3 (5)** | 210 | 166 | 114 | 182 | 127 | 86 | 162 | 329 | 140 |  |  |
| **4 (5)** | 216 | 166 | 94 | 182 | 117 | 100 | 168 | 339 | 154 |  |  |
| **5 (5)** | 218 | 164 | 116 | 184 | 123 | 88 | 170 | 329 | 126 |  |  |
| **6 (4)** | 210 | 166 | 94 | 182 | 127 | 86 | 168 | 339 | 154 |  |  |
| **7 (4)** | 210 | 166 | 94 | 182 | 127 | 100 | 162 | 339 | 140 |  |  |
| **8 (4)** | 216 | 166 | 145 | 178 | 115 | 96 | 166 | 339 | 128 |  |  |
| **9 (3)** | 170 | 164 | 149 | 178 | 117 | 84 | 170 | 331 | 142 |  |  |
| **10 (3)** | 192 | 164 | 139 | 176 | 119 | 86 | 170 | 333 | 134 |  |  |
| **11 (3)** | 210 | 166 | 114 | 182 | 117 | 100 | 168 | 329 | 154 |  |  |
| **12 (3)** | 214 | 164 | 141 | 178 | 121 | 92 | 164 | 314 | 132 |  |  |
| **13 (3)** | 216 | 166 | 145 | 172 | 117 | 96 | 166 | 329 | 128 |  |  |
| **14 (3)** | 216 | 166 | 145 | 178 | 115 | 96 | 170 | 329 | 128 |  |  |
| **15 (3)** | *Q* | 154 | 147 | 176 | 117 | 92 | 168 | 314 | 134 |  |  |
| **16 (3)** | *Q* | 160 | 139 | 182 | 123 | 90 | 170 | 329 | 132 |  |  |
| **17 (2)** | 226 | 164 | 116 | 184 | 123 | 96 | 164 | *Q* | 128 |  |  |
| **18 (1)** | 165 | 164 | 145 | 184 | 119 | 94 | 168 | 333 | 136 |  |  |
| **19 (1)** | 186 | 160 | *Q* | 182 | 119 | 96 | 164 | *Q* | 132 |  |  |
| **20 (1)** | 192 | 164 | 130 | 172 | 123 | 86 | 170 | 318 | 140 |  |  |
| **21 (1)** | *Q* | 166 | 114 | 182 | 119 | 86 | 162 | 329 | 140 |  |  |
| **22 (1)** | *Q* | 166 | 94 | 182 | 119 | 100 | 162 | 339 | 140 |  |  |
| **23 (1)** | 210 | 166 | *Q* | 182 | 117 | 100 | 168 | *Q* | 156 |  |  |
| **24 (1)** | 216 | 164 | 94 | 180 | *Q* | 100 | 168 | 339 | 140 |  |  |
| **25 (1)** | 220 | 164 | *Q* | 178 | 127 | 86 | 162 | 316 | 132 |  |  |
| **26 (1)** | 226 | 164 | 145 | *Q* | 123 | 96 | 164 | 339 | 128 |  |  |

| **Colony** | ***Ne*2** | **(*p* = 1.79 × 10^–3^)** | |  |  |  |  |  |  |  |  |
| --- | --- | --- | --- | --- | --- | --- | --- | --- | --- | --- | --- |
|  |  |  |  |  |  |  |  |  |  |  |  |
| **Locus** | **Eb25** | **Eb51** | **Lp2** | **Lp4** | **Lp14a** | **Lp38** |  |  |  |  |  |
|  |  |  |  |  |  |  |  |  |  |  |  |
| **Queen** | 147 | 126 | 123 | 119 | 136 | 142 |  |  |  |  |  |
|  | 147 | 126 | 123 | 123 | 140 | 148 |  |  |  |  |  |
|  |  |  |  |  |  |  |  |  |  |  |  |
| **Patriline (*n*)** |  |  |  |  |  |  |  |  |  |  |  |
| **1 (22)** | 151 | 124 | 123 | 135 | 172 | 134 |  |  |  |  |  |
| **2 (15)** | 155 | 114 | 123 | 135 | 172 | 148 |  |  |  |  |  |
| **3 (13)** | 161 | 126 | 127 | 121 | 136 | 144 |  |  |  |  |  |
| **4 (11)** | 145 | 112 | 123 | 149 | 136 | 130 |  |  |  |  |  |
| **5 (9)** | 143 | 128 | 119 | 113 | 136 | 142 |  |  |  |  |  |
| **6 (5)** | 145 | 128 | 123 | 113 | 136 | 140 |  |  |  |  |  |
| **7 (5)** | 159 | 112 | 125 | 115 | 138 | 138 |  |  |  |  |  |
