# Supplementary material for: The Evolution of Extreme Polyandry in Social Insects: Insights from Army Ants
Source: PLoS One. 2014 Aug 21;9(8):e105621. doi: 10.1371/journal.pone.0105621 (PMC4140799; doi:10.1371/journal.pone.0105621)
Supplement: Protocol S1 — Microsatellite isolation protocol for Labidus praedator and cross-amplification tests. (DOC) [file pone.0105621.s007.doc]

Protocol S1. Microsatellite isolation protocol for *Labidus praedator* and cross-amplification tests and genotyping of the five Neotropical army ant species *Eciton mexicanum*, *E. vagans*, *L. coecus*, *L. praedator* and *Nomamyrmex esenbeckii*.

Genomic DNA was extracted from 18 workers (after removing gasters) of three *L. praedator* colonies (6 workers per colony) using a phenol-chloroform method with 2% cetyl-trimethyl-ammonium-bromide (CTAB) lysis buffer and 100 µg proteinase k (Diagonal) [1]. Approximately 10 µg DNA were digested with *Rsa*I enzyme (Fermentas) in a volume of 30 µl for 2 h at 37°C prior to the ligation of SuperSNX linkers (T_4_ DNA ligase, Fermentas) by incubating 3 h at 37°C. Subsequently 1 µM biotinylated CT-oligos (Metabion) were hybridized to the ligated fragments by using the polymerase chain reaction (PCR) program *OligoHyb* in a thermocycler [2]. After adding 5 mg magnetic streptavidin beads (Dynal), microsatellite containing DNA was enriched using a magnetic particle concentrator. DNA was recovered by PCR and ligated into pCR®2.1-TOPO® plasmid vectors conferring ampicillin (*amp*) resistance (TOPO TA Cloning® Kit, Invitrogen™). Plasmids were transformed into *amp* sensitive TOP 10 Competent *Escherichia coli* cells (Invitrogen™) and positive clones selected by growth on *amp* (50µl/ml) Agar plates. A total of 48 microsatellite containing fragments could be amplified and sequenced (MWG-Biotech) from positive *E. coli* clones using M13 primers. Primers were designed to match flanking regions of the microsatellites and melting temperatures of *T*_m_ ~ 60°C using the software Primer 3 [3].

Of the 48 microsatellites, five (Lp2, Lp4, Lp14a, Lp30 and Lp38) proofed to be polymorphic in our *L. praedator* samples (Table S1). These five primers and those designed for two other army ant species [4,5] were tested for cross-amplification in our five study species, ending up with a total of 14 suitable microsatellites, which were fluorescence dye labelled (forward primers labelled with FAM, HEX or TET) and used for subsequent genotyping (Table S2). All primer test PCRs were performed using 200 ng of CTAB-extracted template DNA pools of six to 18 individuals (from one to three colonies) and an annealing temperature gradient of *T*_a_ reaching from 50°C to 58°C, and run in a QIAxcel gel electrophoreses (Qiagen). Final PCR cocktails contained 2 µl of 5% Chelex extracted [6] template, 1 × PCR multiplex buffer (Promega) and 2 pmol of each primer (4 pmol for Eb10 primers in *L. praedator*) in total reaction volumes of 10 µl. Lp and Eb primers were multiplexed in pools of two to four primers per PCR (only Eb10 and Lp30 for *L. praedator*, Lp2 for *E. vagans*, and DmoD were run separately). After 2 min (3 min for DmoD) denaturation at 95°C PCR reactions ran in a thermocycler for 35 cycles of 30 s (40 s for DmoD) at 95°C, 30 s at 54°C (40 s at 51°C for DmoD), 1 min at 72°C followed by a final extension of 10 min (5 min for Lp primers) at 72°C and subsequent fragment length determination, allele calling and scoring (see main text).

**References**

1. Sambrook J, Russell DW (2001) Molecular cloning: a laboratory manual. 3rd ed. Cold Spring Harbor: Cold Spring Harbor Laboratory Press.

2. Glenn TC, Schable NA (2005) Isolating microsatellite DNA loci. Methods Enzymol 395: 202–222. doi:10.1016/S0076-6879(05)95013-1.

3. Rozen S, Skaletsky H (2000) Primer3 on the WWW for general users and for biologist programmers. Methods Mol Biol 132: 365–386.

4. Kronauer DJC, Boomsma JJ, Gadau J (2004) Microsatellite markers for the driver ant *Dorylus* (*Anomma*) *molestus*. Mol Ecol Notes 4: 289–290. doi:10.1111/j.1471-8286.2004.00645.x.

5. Denny AJ, Franks NR, Edwards KJ (2004) Eight highly polymorphic microsatellite markers for the army ant *Eciton burchellii*. Mol Ecol Notes 4: 234–236. doi:10.1111/j.1471-8286.2004.00627.x.

6. Walsh PS, Metzger DA, Higuchi R (1991) Chelex-100 as a medium for simple extraction of DNA for Pcr-based typing from forensic material. Biotechniques 10: 506–513.
